# STAT1, IGF1, RAC1, and MDM2 Are Associated with Recurrence of Giant Cell Tumor of Bone

**DOI:** 10.1155/2018/4564328

**Published:** 2018-01-31

**Authors:** Shuxin Chen, Zepeng Du, Bingli Wu, Huiyang Shen, Chunpeng Liu, Xueli Qiu, Yufeng Zhang, Liyan Xu, Enmin Li, Zhigang Zhong

**Affiliations:** ^1^Department of Orthopedic Surgery, Shantou Central Hospital, Affiliated Shantou Hospital of Sun Yat-sen University, Shantou 515041, China; ^2^Department of Pathology, Shantou Central Hospital, Affiliated Shantou Hospital of Sun Yat-sen University, Shantou 515041, China; ^3^Department of Biochemistry and Molecular Biology, Shantou University Medical College, Shantou 515041, China; ^4^Institute of Oncologic Pathology, Shantou University Medical College, Shantou 515041, China

## Abstract

**Background:**

In our previous study, mouse double minute 2 homolog (MDM2), insulin-like growth factor 1 (IGF1), signal transducer and activator of transcription 1 (STAT1), and Rac family small GTPase 1 (RAC1) were correlated with the recurrence of giant cell tumor of bone (GCT). The aim of this study is to use a large cohort study to confirm the involvement of these four genes in GCT recurrence.

**Methods:**

The expression of these four genes was detected and compared between GCT patients with or without recurrence. The correlation between the expression of these four genes and clinical characteristics was evaluated. Protein-protein interaction (PPI) network was constructed for functional enrichment analysis.

**Results:**

It showed that the expression levels of MDM2, IGF1, STAT1, and RAC1 in GCT patients with recurrence were significantly higher than those in GCT patients without recurrence (*P* < 0.05). Multivariate logistic regression analysis suggested that several clinical characteristics may influence prognosis. A PPI network was constructed using the four genes as hub genes. Functional enrichment analysis showed that this network involves many important biological progress mediated by these four genes, including immune response.

**Conclusion:**

MDM2, IGF1, STAT1, and RAC1 are associated with GCT recurrence, which might serve as biomarkers for GCT recurrence.

## 1. Introduction

Giant cell tumor of bone (GCT) is an aggressive osteolytic tumor with a high rate of recurrence. Recurrence after surgery treatment has been reported in about 50% of GCT patients. To date, surgery including wide resection and curettage is the major approach used for the treatment of GCT. While wide resection significantly reduces the recurrence of GCT [1], it destroys bone structure and limits joint function. In addition, the histological origin of GCT is not clear and the biological behavior of GCT is complex and varied, which causes difficulties for both surgical planning and the evaluation of the efficacy of surgery for GCT. Therefore, accurate evaluation of the biological behavior of GCT is important for the treatment of GCT. However, the traditional evaluation of GCT behavior, which is based on the histological classification proposed by Jaffe [2], is insufficient to predict tumor progression. The latter evaluation method, which is based on imaging examination results [3] and clinical stage [4], is still controversial. Currently, no efficient biomarkers are used to evaluate the biological behavior of GCT.

There is increasing evidence suggesting that mouse double minute 2 homolog (MDM2), insulin-like growth factor 1 (IGF1), signal transducer and activator of transcription 1 (STAT1), and Rac family small GTPase 1 (RAC1) are involved in tumor progression. MDM2 is considered as a negative regulator of p53 [5]. Amplification of MDM2 genes and/or enhanced expression of MDM2 is observed in a variety of tumors including GCT [6]. IGF-1, which regulates cell proliferation, plays a key role in osteoblast proliferation and bone formation [7]. STAT1 is a STAT family member and is associated with human breast cancer, melanoma, leukemia, and lymphoma cancers [8]. RAC1 is also involved in tumor invasion, metastasis, and angiogenesis [9]. However, it is currently unclear whether these four genes are associated with GCT recurrence.

Previously, we evaluated differentially expressed genes and analyzed recurrence-related subpathways in the recurrent GCT patients. Based on microarray results and subpathway analyses, four genes (IGF1, MDM2, STAT1, and RAC1) were identified to play an important role in GCT of bone recurrence [10]. In this study, the aim is to verify, by immunohistochemistry, the expression of IGF1, MDM2, STAT1, and RAC1 and investigate their potential relationship with GCT recurrence, in a large cohort of patients with a giant cell tumor of the bone.

## 2. Materials and Methods

### 2.1. Specimens

A total of 75 formalin-fixed, paraffin-embedded GCT tissue blocks were included in the present study. These specimens were obtained from surgically dissected bone tissues from 75 GCT patients who were treated, between January 2000 and June 2012, in the Department of Orthopedics, Shantou Central Hospital affiliated with Zhongshan University. The diagnosis of GCT was confirmed based on histopathological examination by pathologists in the Department of Pathology, Shantou Central Hospital. The 75 primary GCT cases included both postoperative cases with recurrence (*n* = 20) and nonrecurrence (*n* = 55). The mean age of the 36 male and 39 female patients was 31 years (15–65 years). The clinical characteristics of these patients are shown in Table 1. All human studies have been approved by The Institutional Review Board of Shantou Hospital of Zhongshan University and have been performed in accordance with the ethical standards laid down in the 1964 Declaration of Helsinki and its later amendments. All persons gave their informed consent prior to their inclusion in the study.

### 2.2. Immunohistochemistry (IHC)

Mouse anti-human monoclonal antibodies against MDM2, IGF1, STAT1, and RAC1 were purchased from DAKO. Immunohistochemistry SP kits were purchased from Fuzhou Maixin Company. IHC was conducted on paraffin sections using the SP kit according to the manufacturer's instructions. Slides were stained using DAB with hematoxylin contrast staining. The negative controls were incubated with PBS to substitute for the primary antibody.

IHC results were scored based on both staining intensity (no staining, score = 0; weak staining, score = 1; medium staining, score = 2; and strong staining, score = 3) and the percentage of positively stained cells (≤5%, score = 0; 6%~25%, score = 1; 26~50%, score = 2; 51 ~ 75%, score = 3; and >75%, score = 4). The final IHC score for individual tumors was determined by the product of the scores for intensity and percentage of positively stained cells. The final IHC score, for either patients with or patients without recurrence, was determined by the sum of the scores of all individual tumor staining within the group. For example, in three specimens where 25%, 25%, and 50% of tumor cells exhibited medium, weak, and no staining, respectively; the scores, for each individual tumor, were 2 (1 × 2), 1 (1 × 1), and 0 (2 × 0), respectively. The IHC score for the group was 3 (2 + 1 + 0).

### 2.3. Protein-Protein Interaction Network Construction

To explore the correlation between four genes (MDM2, IGF1, STAT1, and RAC1), a protein-protein interaction (PPI) network was constructed by the method as we described before [11]. Briefly, the protein interaction data validated by experiment was obtained from BioGRID (https://thebiogrid.org/) and HPRD (http://www.hprd.org/) to form a parental PPI network. A child PPI was constructed by mapping the four genes as the seed proteins in the parental PPI network to extract their first class interacting proteins by Cytoscape [12].

### 2.4. Functional Enrichment Analysis of PPI Network

Gene ontology (GO) annotation of the four genes, PPI networks, was performed using the ClueGO plugin, which could produce a functional enrichment group GO terms in the form of a network [13]. Only GO terms with a *P* value <0.001 were considered significant. A kappa score reflecting the connection between the GO terms was based on their overlapping genes, which was set to 0.5 as the threshold.

### 2.5. Statistical Analyses

Statistical analyses of the experimental results were conducted using SPSS software (Version 13.0). The correlation between gene expression (of MDM2, IGF1, STAT1, and RAC1) and clinical characteristics was conducted using the Kendall's tau-b test. The association of MDM2, IGF1, STAT1, and RAC1 and tumor recurrence was analyzed using chi-square analysis. Clinical characteristics that may be associated with GCT recurrence were analyzed using logistic regression, and an *α* value of 0.05 was used as a standard for regression analysis.

## 3. Results

### 3.1. MDM2, IGF1, STAT1, and RAC1 Expression in GCT Tissue

**Figure 1:**
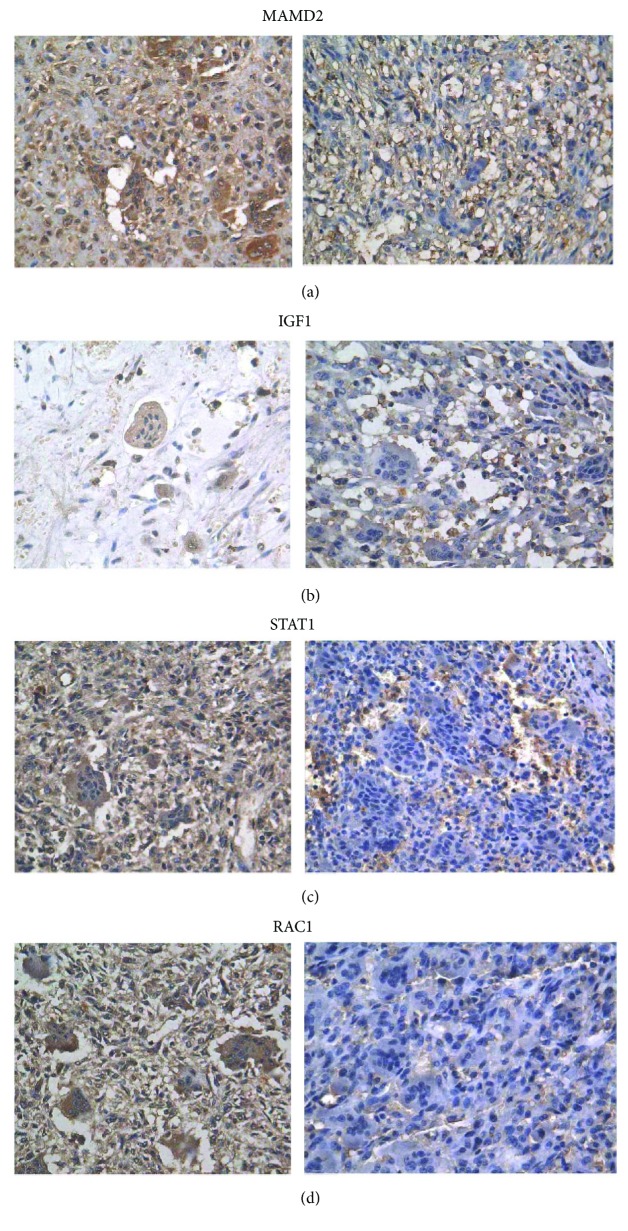
Expression of MDM2, IGF1, STAT1, and RAC1 in GCT tissues (400X, scale bar = 50 *μ*m). (a) Expression of MDM2. Positive staining of MDM2 in the tumor tissue of a recurrent GCT (left). Negative staining of MDM2 in the tissue of a primary GCT from a patient without recurrence (right). (b) Positive staining of IGF1 in the tumor tissue of recurrent GCT (left). Negative staining of IGF1 in the primary tumor tissue of a GCT patient without recurrence (right). (c) Positive staining of STAT1 in the tumor tissue of recurrence GCT (left). Negative staining of STAT1 in the primary tumor tissue of a GCT patient without recurrence (right). (d) Positive staining of RAC1 in the tumor tissue of recurrence GCT (left). Negative staining of RAC1 in the tumor tissue of primary GCT without recurrence (right). Positive staining for all genes was located in the cytoplasm and nuclei of multinucleated giant cells and mononuclear stromal cells.

Immunohistochemistry was performed for MDM2, IGF1, STAT1, and RAC1 to determine their expression patterns in GCT patients with recurrence. As shown in Figure 1, the staining of MDM2, IGF1, STAT1, and RAC1 was mainly found in the cytoplasm of GCT tissues, but nuclear staining was also observed in some cells. Both multinucleated giant cells and mononuclear stromal cells were positively stained for these markers. Generally, the staining of MDM2 and RAC1 was stronger than IGF1 and STAT1 staining. The percentage of positively stained cells was similar for these four markers, which exhibited similar and consistent staining patterns.

### 3.2. Expression of MDM2, IGF1, STAT1, and RAC1 and GCT Patients Is Associated with Recurrence

Immunohistochemistry analyses combined with statistical analyses were conducted to confirm the association between the expression of the four genes and GCT recurrence. As shown in Table 2, the expression of MDM2, IGF1, STAT1, and RAC1 in GCT patients with recurrence was significantly higher than that in GCT patients without recurrence (*P* < 0.05). Positive staining of MDM2 was observed in 16 of 55 GCT tissues without recurrence and 14 of 20 GCT tissues with recurrence. A chi-square test demonstrated that the expression of MDM2 in GCT tissues with recurrence was significantly higher than that in GCT tissues without recurrence (*P* = 0.012) (Table 2). Positive staining for IGF1 was observed in 19 of 55 GCT tissues without recurrence and 14 of 20 GCT tissues with recurrence, with chi-square test suggesting that the expression of IGF1 in GCT tissues with recurrence was significantly higher than that in GCT tissues without recurrence (*P* = 0.033) (Table 2). Positive staining for STAT1 was observed in 15 of 55 GCT tissues without recurrence and 12 of 19 GCT tissues with recurrence. The chi-square test suggested that the expression of STAT1 in GCT tissues with recurrence was significantly higher than that in GCT tissues without recurrence (*P* = 0.026) (Table 2). Positive staining for RAC1 was observed in 15 of 55 GCT tissues without recurrence and 14 of 20 GCT tissues with recurrence, and chi-square test suggested that the expression of RAC1 in GCT tissues with recurrence was significantly higher than that in GCT tissues without recurrence (*P* = 0.007) (Table 2).

### 3.3. Expression of MDM2, IGF1, STAT1, and RAC1 Is Not Associated with Clinical Characteristics of GCT Patients with Recurrence

Association of the expression of MDM2, IGF1, STAT1, and RAC1 and clinical characteristics such as age, gender, Campanicci grade, pathological fracture, and lung metastasis was also analyzed using the Kendall's tau-b test. No significant association was observed between the expression of the four markers and any clinical characteristics, including age, gender, Campanicci grade, pathological fracture, and lung metastasis.

Furthermore, clinical characteristics including gender, age, tumor duration, tumor location, pathologic fracture, and surgical procedures, which may influence prognosis, were analyzed using multivariate logistic regression analysis. No clinical characteristics were identified to be significantly associated with GCT recurrence (Table 3).

### 3.4. PPI Network and Functional Enrichment

**Figure 2:**
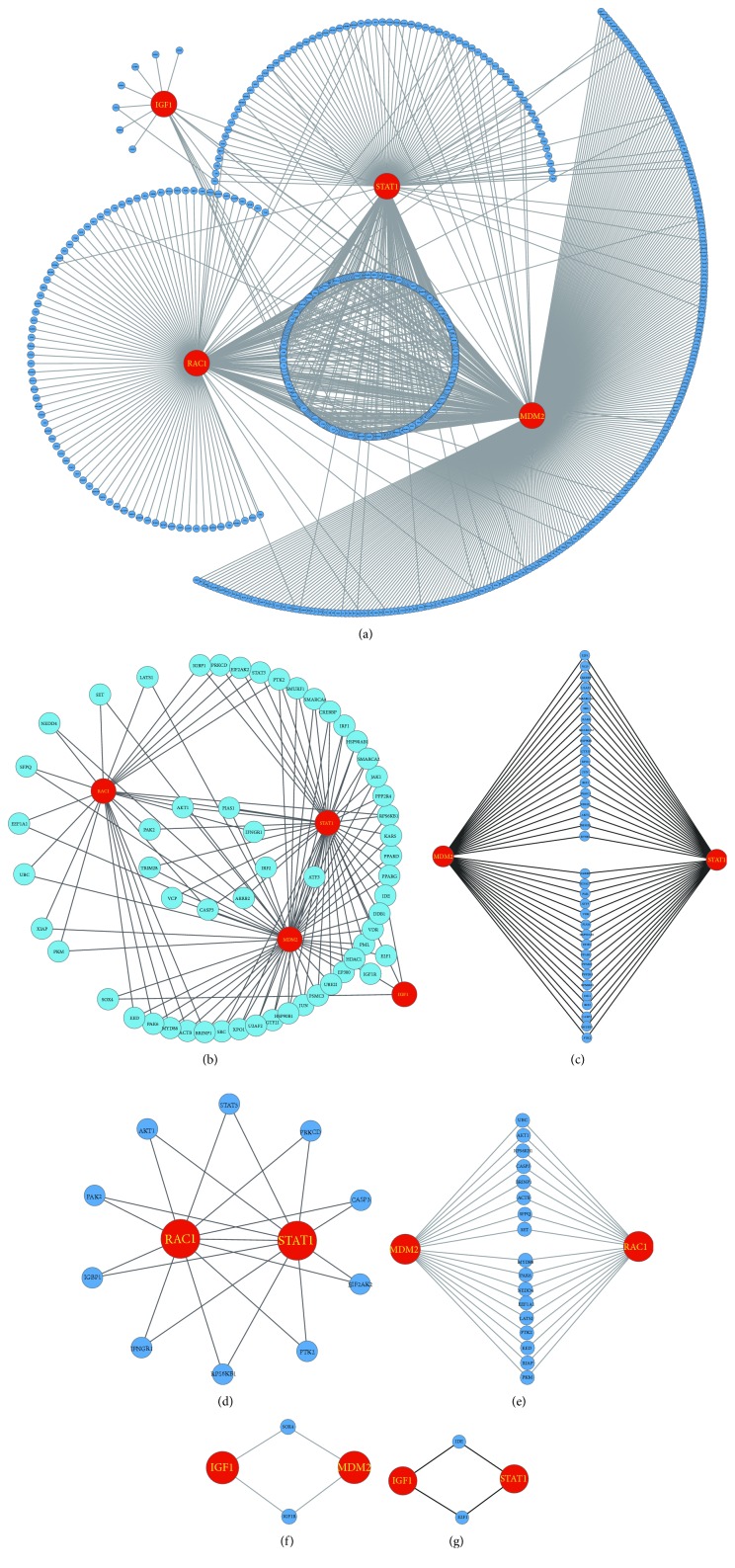
The protein-protein interaction network for MDM2, IGF1, STAT1, and RAC1. (a) MDM2, IGF1, STAT1, and RAC1 are hub proteins in the PPI network as they have hundreds of interacting proteins. (b) MDM2, IGF1, STAT1, and RAC1 are connected by at least two or more shared interacting proteins. (c–g) The interactions of every two genes between MDM2, IGF1, STAT1, and RAC1.

In order to gain insight into how these MDM2, IGF1, STAT1, and RAC1 are connected, a full screen of their interacting proteins would provide important clues of their links and functions. The PPI network in this study contains 768 nodes (proteins) and 830 edges (interactions) as shown in Figure 2(a), suggesting these four genes are hub proteins in the cellular network through the interactions with hundreds of their interacting proteins. To better illustration, only the direct interaction among the four genes was shown (Figures 2(b)–2(g)). We found that MDM2, IGF1, STAT1, and RAC1 are connected with at least two or more shared interacting proteins. RAC1 and STAT1 could interact directly.

**Figure 3:**
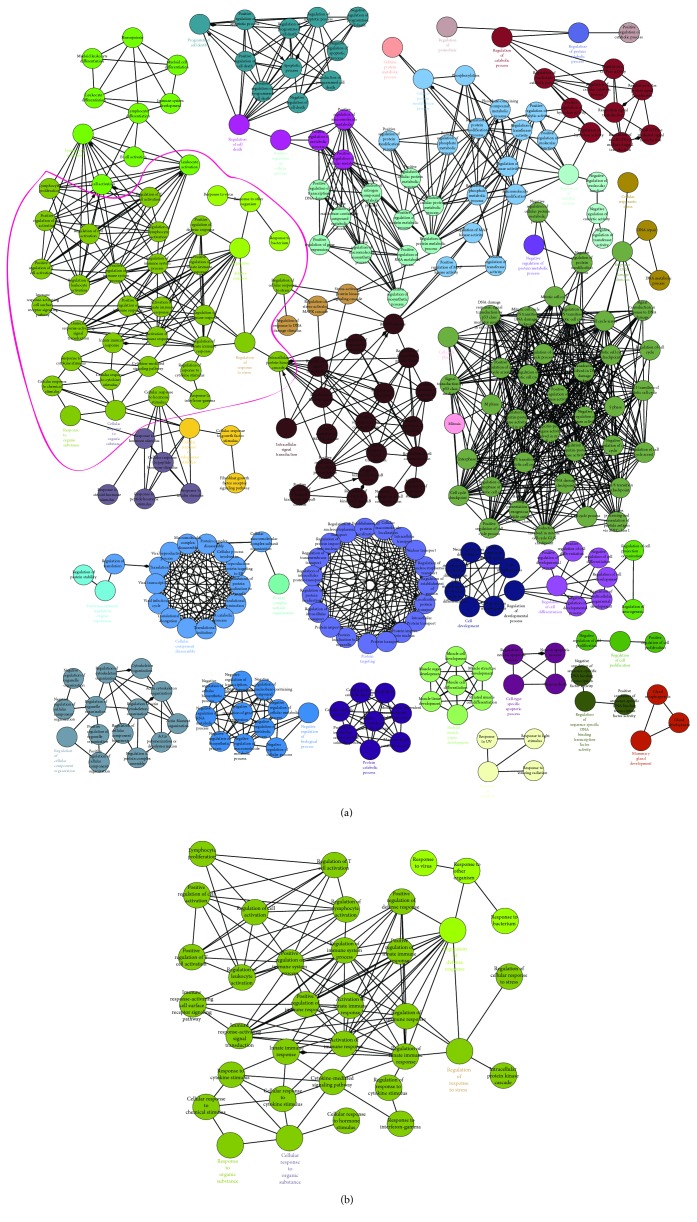
Functional enrichment analysis of the PPI network of MDM2, IGF1, STAT1, and RAC1. (a) 290 significant GO terms were generated by the functional enrichment analysis of the four genes, PPI network. GO terms are connected when they shared enriched genes above the threshold of kappa score. (b) The group of immunity-related terms, as indicated in the pink circle in (a).

A map containing 290 significant GO terms was generated by the functional enrichment analysis of the four genes, PPI network. In this map, the nodes represent as the protein-enriched GO terms, with the edges connecting the GO terms indicative of proteins shared between GO terms (Figure 3(a)). Several GO terms that were related to known functions were found, such as “intracellular signal transduction,” “cell cycle phase,” and “cell development.” Moreover, we found a big group of immunity-related terms, including “regulation of immune system process,” “activation of immune response,” “immune response-activating signal transduction,” “positive regulation of immune response,” “immune response-activating cell surface receptor signaling pathway,” and “regulation of innate immune response” (Figure 3(b)). These results suggested that the four genes involved in GCT recurrence might participate in immune response. The significant immune-related-enriched functional terms are listed in Table 4.

## 4. Discussion

In a previous study, we compared the expression profiles, based on microarray data, of a large number of genes between GCT with and GCT without recurrence [10] and identified six signaling pathways that may play important roles in the development of GCT. In the present study, we further analyze whether the differentially expressed genes STAT1, IGF1, RAC1, and MDM2 are associated with GCT recurrence.

Based on the IHC results, significantly higher expression levels of MDM2, IGF1, STAT1, and RAC1 are found in the patients with recurrent GCT, compared to primary patients, which is consistent with our previous microarray study [10]. Subsequent statistical analyses suggested that these four genes are closely correlated with GCT recurrence, whereas no significant association with clinical characteristics, such as age, gender, Campanicci grade, pathological fracture, and lung metastasis. These findings indicate that MDM2, IGF1, STAT1, and RAC1 may serve as potential biomarkers for prediction of GCT patients at a high risk of recurrence. Nevertheless, researches based on the panel of these four genes in human disease have not been reported so far.

Many reports have confirmed that these four genes play important roles in tumors. Upregulation of MDM2 has also been observed in a variety of sarcoma [14]. Previous studies suggest that upregulation of MDM2 is associated with tumor initiation and metastasis of GCT [15]. MDM2 gene amplification in osteosarcoma is often associated with p53 inactivation [16] or osteosarcoma of metastasis and recurrence; we thus propose that MDM2 is closely related to GCT recurrence and may contribute to tumor cell proliferation. Evidence shows that IGF1 promotes tumor cell differentiation and growth through autocrine or paracrine means [17]. IGF1 is also closely associated with bone metabolism and plays a key role in growth factor promotion of osteoblast proliferation and bone formation [7]. In addition to these findings, IGFI is found to function in stimulating osteoblast proliferation and bone formation and inhibiting bone collagen degradation [18]. It has been recently reported that STAT1 expression is upregulated in osteoporotic bone tissue in mice [19]. Chen et al. reported that the expression of STAT1 in circulating monocytes is essential for bone metabolism, suggesting an important role for the STAT1-mediated IFN pathway in osteoporosis [20]. STAT1 plays an important role in bone growth and bone formation [21], which suggests that STAT1 might be involved in the development of GCT, especially tumor invasiveness. RAC1 expression is upregulated in the osteosarcoma cell lines and tissues, and its ectopic expression promotes the proliferation, migration, and invasion of MG-63 cells [22]. It has been reported that a novel ERBB4-PI3K-AKT-FAK-RAC1 pathway associated with an aggressive disease in Ewing sarcoma [23].

This study is the first time to confirm that MDM2, IGF1, STAT1, and RAC1 might link together involved in GCT recurrence. Proteins do not work alone, but interact with other proteins or molecules to perform their specific functions. MDM2, IGF1, STAT1, and RAC1 serve as hub genes in the PPI network. Functional enrichment result shows that this network involves many biological progress, including immune response. In recent years, the immune activity in bone giant cell has attracted more attention. Al-Sukaini et al. found that locally aggressive giant cell lesions are associated with low HLA class 1 antigen expression, low CD8 + T cell infiltration, and high expression of the immune modulator B7-H3 [24]. On the other hand, most of GCT of bone cases can show variable immunoreactivity for CD10 and CD138 [25]. These results suggest that immune response, as well as other biological functions, mediated by MDM2, IGF1, STAT1, and RAC1 through their interacting proteins, might play an important role in GCT recurrence.

In summary, we provide evidence that MDM2, IGF1, STAT1, and RAC1 are associated with GCT recurrence. MDM2, IGF1, STAT1, and RAC1 may serve as biomarkers to predict GCT recurrence. In addition, the molecular mechanism of these markers in the development of GCT in a panel model should also be explored for better use of these markers in the evaluation of the prognosis of GCT.

## Figures and Tables

**Table 1:** Clinical characteristics of the 75 GCT cases included in the present study.

Clinical characteristics	Postoperative recurrence	Nonrecurrence after surgery	Incidence of postoperative recurrence
75	20	55	26.6%

*Gender*			
Male	9	27	25%
Female	11	28	28.2%

*Tumor location*			
Distal femur	9	23	28.1%
Proximal tibia	2	6	25%
Distal radius	4	4	50%
Proximal humerus	0	6	0
Proximal femur	3	4	42.9%
Other sites	2	12	14.3%

*Pathological fracture*			
Yes	3	12	20%
No	17	43	28.3%

*Campanacci grade*			
I	3	12	20%
II	12	31	27.9%
III	5	12	29.4%

*Surgery*			
Wide resection	6	17	26.0%
Curettage	13	39	25%
Amputation	0	0	0

**Table 2:** Association between expression of MDM2, IGF1, STAT1, and RAC1 and the recurrence of GCT.

	MDM2		IGF1		STAT1		RAC1	
Staining intensity	—	+	—	+	—	+	—	+
Without recurrence	39	16	36	19	41	15	40	15
With recurrence	6	14	6	14	7	12	6	14
*P* value	0.003		0.009		0.006		0.001	

**Table 3:** Logistic regression analysis of the association between clinical characteristics and GCT recurrence.

		Score	Difference	Significance
Variables	Gender	0.783	1	0.376
	Age	0.511	1	0.475
	C grade	0.282	2	0.869
	C grade (1)	0.275	1	0.600
	C grade (2)	0.140	1	0.709
	Pathological fracture	0.078	1	0.781
	Treatment	0.739	1	0.390
	Adjuvant	0.168	1	0.682
	Burr	1.345	1	0.246
	Lung metastasis	2.641	1	0.104

Overall statistics		16.352	10	0.090

**Table 4:** The significant immune-related-enriched functional terms.

Significant GO list	Term name	*P* value corrected with Bonferroni
GO:00713100	Cellular response to organic substance	1.23*E*-28
GO:00072430	Intracellular protein kinase cascade	4.40*E*-27
GO:00100330	Response to organic substance	1.81*E*-26
GO:00192210	Cytokine-mediated signaling pathway	3.80*E*-19
GO:00713450	Cellular response to cytokine stimulus	1.23*E*-17
GO:00801350	Regulation of cellular response to stress	3.38*E*-14
GO:00026820	Regulation of immune system process	7.62*E*-14
GO:00450880	Regulation of innate immune response	8.01*E*-14
GO:00328700	Cellular response to hormone stimulus	9.06*E*-14
GO:00027570	Immune response-activating signal transduction	5.49*E*-10
GO:00026840	Positive regulation of immune system process	1.02*E*-09
GO:00022530	Activation of immune response	3.26*E*-09
GO:00607590	Regulation of response to cytokine stimulus	6.86*E*-09
GO:00508650	Regulation of cell activation	1.11*E*-08
GO:00517070	Response to other organism	2.13*E*-08
GO:00507780	Positive regulation of immune response	2.14*E*-08
GO:00512490	Regulation of lymphocyte activation	4.19*E*-08
GO:00450890	Positive regulation of innate immune response	1.86*E*-07
GO:00024290	Immune response-activating cell surface receptor signaling pathway	2.62*E*-07
GO:00508700	Positive regulation of T cell activation	2.07*E*-06
GO:00508670	Positive regulation of cell activation	3.30*E*-06
GO:00313490	Positive regulation of defense response	3.42*E*-06
GO:00096150	Response to virus	4.46*E*-06
GO:00466510	Lymphocyte proliferation	1.73*E*-05
GO:00096170	Response to bacterium	2.17*E*-05
